# Surgical Task Shifting in Sub-Saharan Africa

**DOI:** 10.1371/journal.pmed.1000078

**Published:** 2009-05-19

**Authors:** Kathryn Chu, Peter Rosseel, Pierre Gielis, Nathan Ford

**Affiliations:** 1 Médecins Sans Frontières, Johannesburg, Gauteng, South Africa; 2 Médecins Sans Frontières, Brussels, Belgium

## Abstract

Kathryn Chu and colleagues discuss some of the experiences of surgical task shifting to date, and outline lessons from task shifting in the delivery of HIV/AIDS care.

**Summary Points**

- Surgically treatable problems account for a significant proportion of disease burden in resource-limited settings, but are neglected due to lack of skilled professionals, adequate infrastructure and equipment, and the perception that surgical services are complex and expensive.
- In the absence of trained surgeons, surgical tasks are often performed by non-specialist physicians and non-physician clinicians. While evaluations have proven the effectiveness of such task shifting, this is often done de facto, with little supervisory or training framework in place.
- As efforts increase to scale up surgical care in the developing world, a number of important lessons from task shifting in the field of HIV/AIDS care could serve to support task shifting in surgery.
- These include clearly defining the limits of task shifting, ensuring adequate training and supervision, providing adequate recognition and remuneration, developing simplified tools and guidelines, ensuring engagement with regulatory bodies, and mobilizing community health workers.

## Introduction

The poor availability of surgical services in developing countries is a long neglected problem that has recently gained attention [1],[2]. Violence, injury, and obstetric emergencies are among leading causes of mortality and morbidity that can be mitigated through surgical intervention. Surgically treatable problems are estimated to account for up to 11% of the world's disability-adjusted life years [1]. In addition to this massive disease burden there are problems that are seriously debilitating (cataract) or stigmatizing (fistula).

Surgical interventions are often viewed as expensive and complex, but many common problems amenable to surgery in resource-limited settings are cost-effective and do not require specialized staff and equipment. The World Health Organization (WHO) has prioritized a list of cost-effective surgical interventions for developing countries including emergency care of trauma, obstetrical complications, and acute abdomens as well as elective care of hernias, clubfoot, cataracts, hydroceles, and otitis media [3].

One of the main barriers to surgical care—defined as the safe provision of pre-operative, operative, and post-operative surgical and anesthesia services—in resource-limited settings is the shortage of trained health workers. Africa accounts for 24% of the global disease burden but only 3% of the global health workforce [4]. The reasons for this are well documented and include inadequate salaries and poor working conditions leading to staff attrition, unwillingness of international donors to support financing for human resources [5], an insufficiency of medical schools [6], and the brain drain of health staff to resource-rich countries [4],[7],[8]. The human resource crisis is most acute at the level of specialists, including surgeons and anesthesiologists [9],[10]. In East Africa, there are 0.25 fully trained surgeons per 100,000 persons compared to 5.69 per 100,000 in the United States [11],[12]. The actual minimum number of surgeons required is unknown.

Given the unlikelihood of even a modest increase in the number of surgeons and anesthesiologists working in Africa in the near future, a number of approaches are being piloted to overcome the skills shortage. These include surgical camps and specialist outreach programs (often supported by international experts) and the mobilization of non-physician clinicians (NPCs) to perform surgical and anesthetic tasks [13]. This latter approach, which involves the shifting of tasks from surgeons and anesthesiologists to non-specialists, has the greatest potential to provide coverage of basic surgical care, especially in rural areas. Task shifting involves the delegation of certain medical responsibilities to less specialized health care workers. In sub-Saharan Africa, task shifting has recently been promoted and formalized to help address the HIV/AIDS epidemic [14]. This paper discusses some of the experiences of surgical task shifting to date, and outlines lessons from task shifting in the delivery of HIV/AIDS care.

## Task Shifting in Surgery

The concept of a surgeon as a university-trained physician is a relatively modern one—prior to 1745 surgeons were still part of the Company of Barbers—but today surgery has become a highly specialized profession. Currently, much of the global surgical workforce is comprised of non-specialist physicians whose only formal surgical training is during medical school. In Uganda, a study of five general hospitals reported that over 5,000 surgical procedures were performed annually by non-specialist physicians [13]. In many countries, where doctors are scarce, certain surgical tasks are delegated to NPCs: almost half of all countries in sub-Saharan Africa use NPCs to perform minor surgical procedures [15]. In Tanzania and Mozambique, 84% and 92% (respectively) of cesarean sections, obstetric hysterectomies, and laparotomies for ectopic pregnancy are performed by NPCs [16],[17]. In Malawi, 90% of cesarean sections at district hospital level are performed by surgical clinical officers with low morbidity and mortality [18]. International organizations have supported task shifting in a number of resource-limited settings (Box 1).

**Box 1. Examples of International Organizations Supporting Surgical Task Shifting**

**Médecins Sans Frontières** has provided a range of MoH-approved training programs, including teaching non-specialist physicians basic surgical skills in Chad (six-month course), and nurse anesthetists in Haiti (15-month course). In conflict settings where formal training was not possible (Angola and Somalia), ad hoc trainings have been provided to nurses so that they could carry out life-saving interventions such as cesarean sections and intestinal resections when no physician was available.

**The International Committee of the Red Cross (ICRC)** supports a MoH-approved surgical training program for non-specialist physicians and nurses in anesthesia in Eritrea. From 1991–1995, ICRC trained non-specialist physicians in Cambodia who were providing most of the surgical care after the Pol Pot regime. ICRC has also supported informal training of physicians and nurses in basic surgery in the Democratic Republic of the Congo and Somalia.

**Serving in Mission (SIM) International** trains nurses in a two-year apprenticeship model to perform operations at Kalukembe District Hospital in Angola (http://www.ceml.net/). This program, which has been running for over 20 years, has not yet been recognized by the MoH, but these nurses have been a critical resource in delivering surgical care in this poor rural district where there are no physicians.

**The Global Health Access Program** (http://www.ghap.org/), working along the Thai/Burmese border in Myanmar, trains non-physician “backpack medics” in skills such as basic resuscitation, surgical stabilization, fracture management, and wound care.

The **Christian Blind Mission** trains NPCs to perform cataract surgery across Africa.

There is resistance to delegating surgical procedures to lower cadres for a number of reasons. Surgery is considered a highly specialized field that requires long years of training: in the US, surgeons complete a five-year surgical residency before operating independently. Indications for surgery are not always straightforward, patient management decisions can be complex, and learning the technical skills required to perform major surgery requires committed trainers. All this leads to a concern that such complex skills and knowledge cannot be adequately transferred in a shortened training course.

While the epidemiology of surgical disease in the developing world is relatively unknown, simple procedures can be life-saving. The most common procedures performed at district hospitals are dilation and curettage and cesarean sections, while the most common general surgical procedures are suturing of wounds and manipulation of fractures [19]. These can be safely managed by non-surgeons. Highly complicated procedures that require the expertise of fully trained surgical specialists are referred to tertiary hospitals.

Evaluations of task shifting in surgery have concluded that NPC cadres are safe and effective. In Mozambique, *técnicos de cirurgia* have supported surgical care in district hospitals since 1989, performing 92% of emergency obstetrical care and 65% of major general surgery [20]. Moreover, because their training can be less expensive and shorter, NPCs are highly cost-effective [4],[15],[21]; by one estimate, operations performed by NPCs cost less than a tenth of those performed by doctors [20].

NPCs are often from the local area, understand local customs and the language, and are more likely to remain in their home country because their training is not internationally recognized: 88% of the *técnicos de cirurgia* trained in Mozambique were still working at district hospitals seven years after their training, while none of the junior doctors who started at the same time remained. In Malawi, 112 orthopedic clinical officers have been trained since 1984 and all have remained in the country [21].

## Lessons from Task Shifting in HIV Care

While these examples are encouraging, task shifting in surgery is for the most part ad hoc. As attention is increasing around the need to address human resource shortages for surgery, the potential for task shifting will likely be encouraged [22],[23]. Task shifting has gained significant attention in the field of HIV/AIDS care, where a growing evidence base is informing policy and practice [14]. Some core principles are emerging that could support its application to surgery.

### 1. Defining the Limits

To promote task shifting in HIV care, WHO developed guidelines that outline over 150 essential tasks for the management of HIV patients, which can be shifted to NPCs and lay workers. The WHO's Clinical Procedures Unit has established a list of surgical tasks that can be safely and effectively performed at the district hospital level [24]. This work needs to be expanded to define a framework for task shifting that would outline which procedures can be safely performed by different health cadres (Table 1). With studies describing mortality rates as high as 5%–10% in developing countries [25]–[27], defining the limits of task shifting is essential to ensure quality of care. This requires innovative testing of new approaches together with rigorous evaluation to build the evidence base for policy. International nongovernmental organizations and academic institutes can support governments in the elaboration and implementation of a priority operational research agenda, establish pilot programs, and evaluate outcomes.

**Table 1:** Examples of surgical tasks that could be performed by different health workers.

Health Cadre	Level of Care	Procedures Performed
Surgeon+anesthesiologist	Tertiary hospital	Neurosurgery; Thoracic surgery; Vascular surgery; Complex orthopedic surgery; Endocrine surgery; Reconstructive surgery; Critical care
General doctor/non-physician clinician with surgical skills+nurse anesthetist	District hospital	Incision and drainage of abscess; Wound debridement; Acute burn care; Skin graft; Circumcision; Hernia repair; Dilation and curettage; Manual placenta extraction; Cesarean section; Exploratory laparotomy for ectopic pregnancy or ovarian torsion; Hysterectomy; Appendectomy; Bowel resection; Stoma creation; Cholecystectomy; Splenectomy; Repair of perforated gastroduodenal ulcer; Limb amputation; Thoracostomy; Closed fracture reduction; Skeletal traction
Community health worker	Primary health center	Pre-hospital transport of trauma victims; Basic wound care management; Referral of surgical disease

### 2. Providing Training, Supervision, and Referral Systems

To ensure adequate quality, reduce burnout, and encourage recognition of responsibilities, the provision of training, supervision, monitoring, and evaluation are all critical. In HIV care, nurses and other NPCs are responsible for direct patient care, while doctors are taking on a supervisory role, often working as part of a mobile team overseeing multiple clinics and providing training and mentorship of complex cases [28]. Similarly, increasing capacity for basic surgical care by NPCs implies the increased need to provide specialists to train and supervise lower cadres and to manage complex cases and complications. Currently, some countries such as Zimbabwe rely on itinerant expatriate surgeons who rotate through district hospitals to assist non-specialist physicians with complex cases and to provide continuing surgical training. This should be formalized in order to provide sustainable support in a systematic manner.

### 3. Ensuring Adequate Recognition and Remuneration

Giving lower cadres more responsibilities is unlikely to be sustainable unless adequate recognition and remuneration is provided. In Mozambique, training of *técnicos de cirurgia* was recognized by government and doctors who welcomed them as colleagues; Malawi, Zambia, and Tanzania also have well established cadres of surgical NPCs that are recognized formally and supported by the Ministries of Health (MoHs) [19]. As has been argued for HIV care, task shifting in surgery should be seen primarily as a way to increase access to care, not to save money, as financial incentives are still required to ensure retention of lower cadres [29].

### 4. Developing Adapted Guidelines

Task shifting in HIV care has been supported by the development of standardized protocols, including simplified clinical guidelines and simplified recording, reporting, and monitoring and evaluation systems. Basic guidelines for surgery are provided by the WHO's Emergency and Essential Surgical Care project, with basic surgical training supported through the Integrated Management of Emergency and Essential Surgical Care toolkit, including a manual for surgical care at the district hospital [30]. Guidelines and protocols for simplification need to be developed for different cadres of health workers if task shifting is to be scaled up. *Primary Surgery* by Maurice King is an invaluable guide for non-surgeons performing surgery in the resource-poor district hospital setting. Similarly, manuals providing practical management of obstetrical emergencies for non-obstetricians working in developing countries exist. These texts should be made broadly available [31],[32].

### 5. Simplification

In HIV care, standardized drug regimens and lab investigations did much to support the simplification of HIV care such that it could be taken on by lower health cadres [33]. Similar approaches are needed for surgery. Minimum standards have been established for safe, effective anesthesia techniques relying on a minimal arsenal of drugs, disposables, and basic equipment, and these should be promoted [34],[35]. Simplified data recording and monitoring and evaluation systems are also needed to provide outcome data to identify areas where further training and supervision is needed.

### 6. Engaging with Regulatory Frameworks and Professional Bodies

Formal recognition by physician professional societies is essential, and the reluctance of professional bodies to support task shifting has been recognized as a barrier to task shifting in HIV care [36]. In Haiti, although the MoH recognized nurse anesthetists as an official cadre, some anesthesiologists still viewed them as assistants and were reluctant to allow them to function independently. Specialists such as surgeons and anesthesiologists should also take leadership roles in the training and accreditation of these lower cadres and help define their responsibilities and limits. In some conflict settings, the MoH ceases to function and there is no regulatory body to provide official recognition of training or new cadres.

### 7. Exploring the Potential for Community Support

Community health workers (CHWs) have recently been engaged to support HIV/AIDS care, where considerable work has been done to define frameworks for engagement [37]. In surgery, CHWs have been successfully trained in the past to recognize diseases requiring surgical intervention, such as hernias and hydroceles. The full potential of CHWs in the provision of surgical care needs to be explored. With proper training and supervision, a minimum package could be administered that might include wound management, basic life support and trauma care, and referral for acute surgical needs such as cesarean section for obstructed labor.

## Ethics of Task Shifting

Shifting clinical responsibilities from higher to lower cadres raises ethical concerns about lowering standards of care. However, while medical ethics clearly insist that doctors provide the best standard of care they can for their patients, public health ethics require health professionals to also consider how to help patients who cannot access care. Health professionals have a duty to ensure that benefits and burdens be fairly balanced, being mindful not just of the fortunate few who get to see a surgeon, but of the invisible majority who never will [38]. In HIV care it was recognized from the outset that if the Western model of care—infectious disease specialists in tertiary hospitals with a multitude of diagnostic and therapeutic options—were to be replicated, then only a fraction of the millions of people in need of treatment in the developing world would benefit, while most of the rest would die. Simplification of protocols and task shifting of key clinical tasks was embraced as a means of ensuring broad benefit and avoiding the creation of “islands of excellence in a sea of under-provision” [39]. Similarly, capacitating lower health cadres to undertake specific surgical tasks aims to maximize benefits while minimizing harm in settings where the unmet need of surgical care is great. The development of a clear, evidence-based framework defining the limits would establish boundaries for what is already happening by default across rural Africa, where surgeons are absent but patients need care.

## Conclusions

The dire shortage of surgeons and anesthesiologists working in Africa means that non-specialist physicians and NPCs provide the majority of surgical services in district hospitals. Efforts should focus on training, supervision, and recognition for these de facto surgeons and anesthesiologists. At the same time, opportunities for further task shifting need to be explored, given the reality that doctors also are in short supply, particularly in rural areas. As CHWs are showing, even people who have not received any formal training in health care can with a minimum of training make an important contribution.

## Abbreviations

CHW: community health worker
MoH: Ministry of Health
NPC: non-physician clinician
